# LncRNA-MEG3 inhibits activation of hepatic stellate cells through SMO protein and miR-212

**DOI:** 10.1038/s41419-018-1068-x

**Published:** 2018-10-03

**Authors:** Fujun Yu, Wujun Geng, Peihong Dong, Zhiming Huang, Jianjian Zheng

**Affiliations:** 1 Departments of Gastroenterology and Hepatology, The First Affiliated Hospital of Wenzhou Medical University, Wenzhou, 325000 China; 2 Department of Anesthesiology, The First Affiliated Hospital of Wenzhou Medical University, Wenzhou, 325000 China; 3 Department of Infectious Diseases, The First Affiliated Hospital of Wenzhou Medical University, Wenzhou, 325000 China; 4 Key Laboratory of Diagnosis and Treatment of Severe Hepato-Pancreatic Diseases of Zhejiang Province, The First Affiliated Hospital of Wenzhou Medical University, Wenzhou, 325000 China

## Abstract

Activation of hepatic stellate cells (HSCs), a pivotal event in liver fibrosis, is considered as an epithelial–mesenchymal transition (EMT) process. Deregulation of long noncoding RNAs (lncRNAs) has been reported to be involved in a series of human diseases. LncRNA-maternally expressed gene 3 (MEG3) functions as a tumor suppressor in cancers and has been shown to play a vital role in EMT process. However, the biological role of MEG3 in liver fibrosis is largely unknown. In this study, MEG3 was reduced in vivo and in vitro during liver fibrosis. Restoring of MEG3 expression led to the suppression of liver fibrosis, with a reduction in α-SMA and type I collagen. Notably, MEG3 overexpression inhibited HSC activation through EMT, associated with an increase in epithelial markers and a reduction in mesenchymal markers. Further studies showed that Hedgehog (Hh) pathway-mediated EMT process was involved in the effects of MEG3 on HSC activation. Smoothened (SMO) is a member of Hh pathway. Using bioinformatic analysis, an interaction between MEG3 and SMO protein was predicted. This interaction was confirmed by the results of RNA immunoprecipitation and deletion-mapping analysis. Furthermore, MEG3 was confirmed as a target of microRNA-212 (miR-212). miR-212 was partly responsible for the effects of MEG3 on EMT process. Interestingly, MEG3 was also reduced in chronic hepatitis B (CHB) patients with liver fibrosis when compared with healthy controls. MEG3 negatively correlated with fibrosis stage in CHB patients. In conclusion, we demonstrate that MEG3 inhibits Hh-mediated EMT process in liver fibrosis via SMO protein and miR-212.

## Introduction

Hepatocellular carcinoma (HCC) and chronic liver diseases (CLDs) such as cirrhosis are a major cause of morbidity and mortality worldwide^1^. Liver fibrosis, a major characteristic of most CLDs, represents the final common pathway of virtually all types of CLDs^2^. Liver fibrosis, characterized by an excessive accumulation of extracellular matrix (ECM) proteins, is a wound-healing response of the liver to various liver injuries such as hepatitis B virus infection^3^. Persistence of liver injury resulted in the formation of crosslinked scars, which is associated with the activation of hepatic stellate cells (HSCs), a major ECM-producing cell^4^. Activated HSCs will become proliferative and fibrogenic, contributing to liver fibrosis progression. Therefore, understanding the mechanisms of activation of HSCs is required for the development of effective anti-fibrotic treatment.

Epithelial–mesenchymal transition (EMT) is a process where epithelial cells gradually express mesenchymal signatures. EMT process has been demonstrated to be involved in HSC activation^5^. Choi et al. found that EMT process in HSC activation is modulated by Hedgehog (Hh) pathway^6^. The main members of Hh family are Sonic Hh, Patched (PTCH), and the Smoothened (SMO) and GLI family zinc finger (GLI). When Hh ligands bind to their receptor PTCH, Hh pathway is activated. The inhibitory effects of PTCH1 on SMO are relieved. Then, SMO translocates to the cilium where it promotes the nuclear localization of the transcription factors GLI1, GLI2, and GLI3, which control the expression of Hh-target genes.

Long noncoding RNAs (lncRNAs), greater than 200 nt, regulate gene expression with little or no protein-coding capacity. LncRNAs modulate gene expression through diverse molecular mechanisms, including chromatin modification, transcriptional regulation, and posttranscriptional regulation. It has been reported that lncRNAs are frequently deregulated in a variety of human diseases and aberrant lncRNAs are involved in disease progression^7,8^. In addition, a growing body of evidence suggests the involvement of lncRNAs in pivotal biological processes such as proliferation and apoptosis^9^. Recent studies have demonstrated that lncRNAs act as regulators of HSC activation in liver fibrosis^10–12^.

Maternally expressed gene 3 (MEG3), a lncRNA, locates in the imprinted DLK1–MEG3 locus on human chromosome 14q32.3 region. MEG3 is expressed in many normal tissues and has been demonstrated to act as a tumor suppressor in various cancers^13–15^. It is becoming increasingly clear that MEG3 plays a crucial role in regulation of EMT process in human diseases^16,17^. Recently, increasing evidence shows the inhibitory effects of MEG3 on liver fibrosis^18^. However, the understanding of the role of MEG3 in liver fibrosis remains limited.

## Results

### Downregulation of MEG3 in fibrotic liver tissues and activated HSCs

**Fig. 1:**
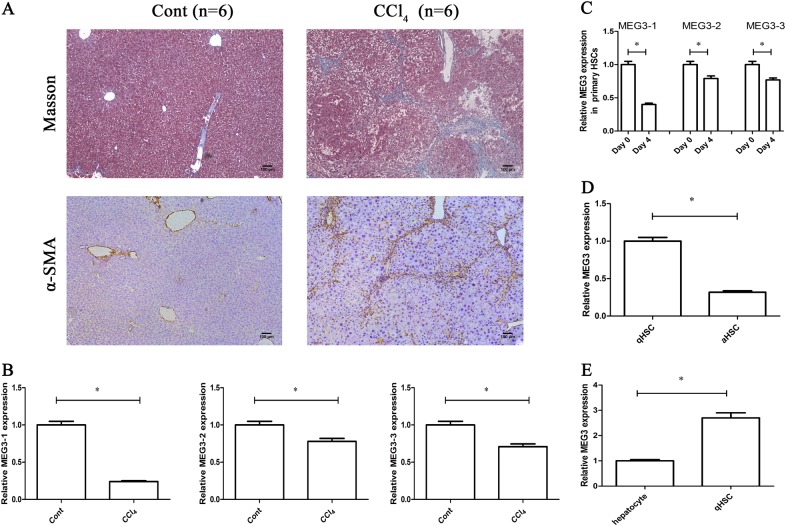
Downregulation of MEG3 in liver fibrosis. **a** Collagen and α-SMA were analyzed in CCl_4_ mice by Masson staining and immunohistochemistry, respectively. Scale bar, 100 μm. **b** MEG3-1, MEG3-2, and MEG3-3 expressions were detected by qRT-PCR in CCl_4_ mice. **c** Expressions of MEG3-1, MEG3-2, and MEG3-3 were analyzed in primary HSCs at Day 0 and Day 4. Primary HSCs were isolated from the livers of healthy mice. **d** MEG3 was analyzed in primary HSCs isolated from oil- or CCl_4_-treated mice. **e** MEG3 was analyzed in primary HSCs and primary hepatocytes from the livers of healthy mice. ^*^P < 0.05

In experimental rodent models, carbon tetrachloride (CCl_4_) is the most commonly used hepatotoxic reagent to produce a typical liver fibrosis model^19^. As shown by Masson staining, collagen expression was significantly induced by CCl_4_ (Fig. 1a). Immunohistochemical images also demonstrated that CCl_4_ treatment resulted in an increase in α-SMA protein level (Fig. 1a). Next, MEG3 expression was detected in mice after CCl_4_ treatment. MEG3 isoforms, which could be found in NCBI (National Center for Biotechnology Information) database, are MEG3 transcript variant 1 (NR_003633.3), MEG3 transcript variant 2 (NR_027651.2) and MEG3 transcript variant 3 (NR_027652.1) (Fig.S1A). Quantitative real-time polymerase chain reactionq (qRT-PCR) analysis showed that the expressions of all MEG3 variants were reduced in fibrotic livers compared with the control livers (Fig. 1b). In comparison with MEG3 variant 2 (MEG3-2) and variant 3 (MEG3-3), MEG3 variant 1(MEG3-1) is obviously reduced in CCl_4_ mice. As shown in Fig.S1B, lower MEG3-1 was found in primary HSCs isolated from CCl_4_ mice compared with MEG3-2 and MEG3-3. Freshly isolated HSCs are known to be activated during culture days, with an increase in mesenchymal phenotype markers and a reduction in quiescent phenotype markers^5^. Then, primary HSCs were isolated from the livers of healthy mice and cultured up to 4 days. Our results additionally showed that lower MEG3-1 was found at Day 4 compared with MEG3-2 and MEG3-3 (Fig. 1c). Thus, MEG3-1 was selected for the following analysis. To confirm that MEG3 expression was downregulated in activated HSCs (aHSCs) in vivo, primary HSCs were isolated from oil- or CCl_4_-treated mice. Expression of MEG3 was significantly lower in aHSCs from CCl_4_ mice compared with that in quiescent HSCs (qHSCs) from healthy controls (Fig. 1d). These results suggest that MEG3 was reduced during HSC activation. Interestingly, there are 15 human MEG3 transcript variants in NCBI database. Due to the major ECM proteins in liver produced by aHSCs, MEG3 expression was examined in transforming growth factor-β1 (TGF-β1)-treated LX2 cells. All MEG3 variants were downregulated in cells after TGF-β1 treatment and several MEG3 variants were reduced at least 70% (Fig.S1C). Therefore, the universal primers for amplifying all human MEG3 variants were designed and used. Our results indicated that MEG3 was obviously reduced in LX2 cells in comparison with primary hepatocytes (Fig. S1D). MEG3 expression was additionally detected in primary HSCs and primary hepatocytes isolated from healthy mice. MEG3 was higher in primary HSCs than that in primary hepatocytes (Fig. 1e). However, MEG3 was reduced in aHSCs compared with primary hepatocytes (Fig. S1E). These data suggest that MEG3 participates in HSC activation.

### MEG3 functions as a potential liver fibrosis suppressor in vivo

**Fig. 2:**
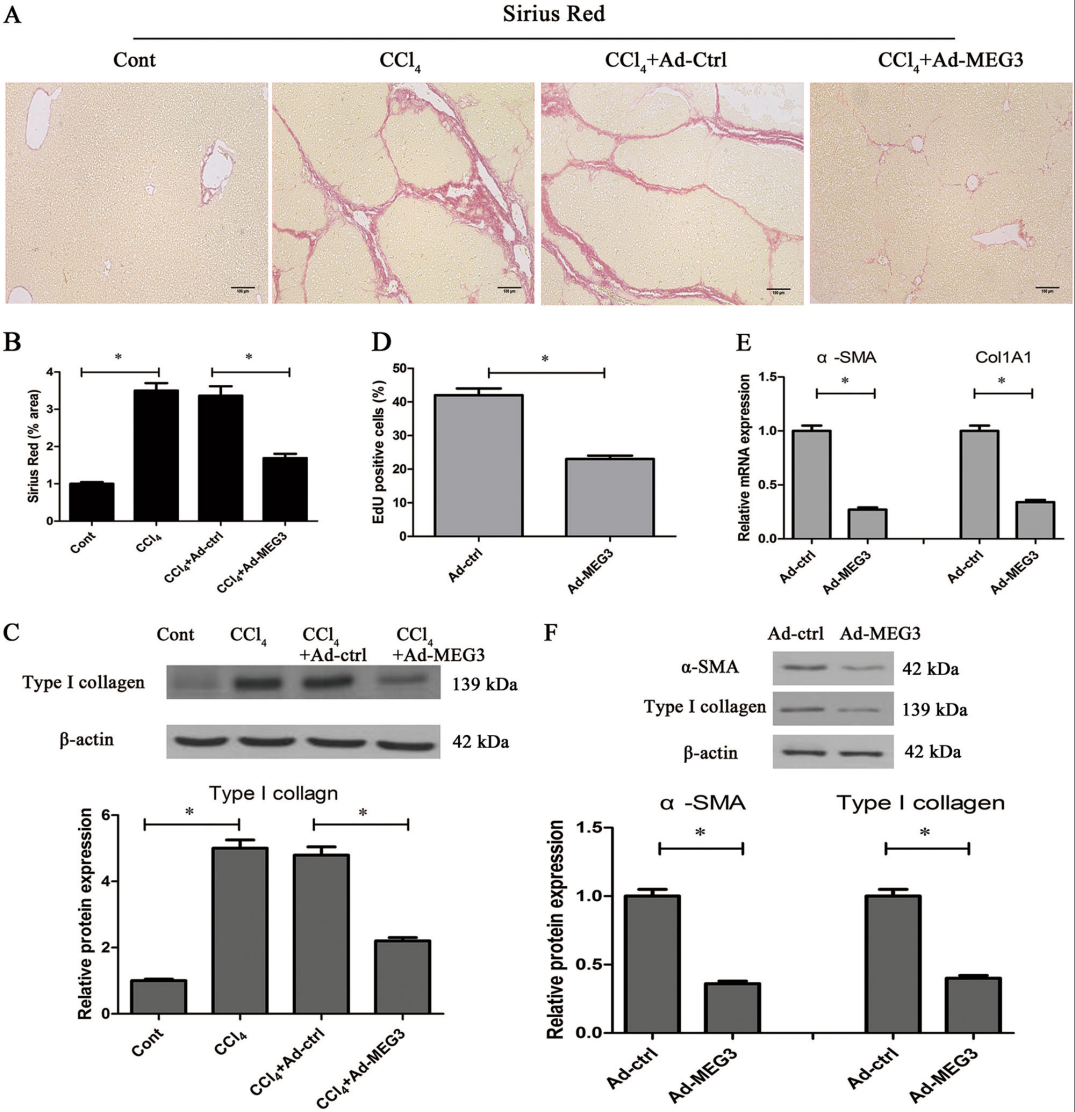
Upregulation of MEG3 inhibits liver fibrosis in vivo and in vitro. **a**, **b** Collagen deposits were analyzed by Sirius Red staining. *n* = 6 mice per group. Scale bar, 100 μm. **c** Type I collagen level was suppressed by MEG3 overexpression in vivo. **d** HSC proliferation was analyzed by EdU assays in primary HSCs at Day 0 after Ad-MEG3 treatment for 48 h. The mRNA (**e**) and protein (**f**) expressions of α-SMA and Col1A1 were analyzed in primary HSCs at Day 0 after Ad-MEG3 treatment for 48 h. ^*^P < 0.05

Adenoviral vectors expressing MEG3 (Ad-MEG3) was constructed and transferred into CCl_4_-mice to explore whether MEG3 has an antifibrotic role in liver fibrosis. Ad-MEG3 led to a significant increase in MEG3 expression in the livers of CCl_4_ mice as well as isolated primary HSCs from CCl_4_ mice (Fig. S2A and Fig. S2B). Sirius Red staining showed that MEG3 overexpression resulted in the suppression of CCl_4_-induced collagen level (Fig. 2a, b). Moreover, hydroxyproline content showed that CCl_4_-induced hydroxyproline level was inhibited by MEG3 (Fig. S2C). Consistent with these results, analysis of immunoblot indicated that increased type I collagen induced by CCl_4_ was blocked down by MEG3 (Fig. 2c). The similar results were found in isolated primary HSCs from CCl_4_ mice after Ad-MEG3 treatment (Fig. S2D). However, in CCl_4_ mice, Ad-MEG3 had no effect on ALT or AST value (Fig. S2E and Fig. S2F). Our results suggest an anti-fibrotic role of MEG3 in liver fibrosis.

### Upregulation of MEG3 reduces activation of HSCs in vitro

HSC activation is characterized by increased cell proliferation, accumulated ECM and enhanced α-SMA synthesis^20^. To investigate the anti-fibrotic role of MEG3 in HSC activation, primary HSCs at Day 0 were transduced with Ad-MEG3. As shown by Fig. S3A, delivery of MEG3 effectively induced an elevation in MEG3 expression. Analysis of Edu assays indicated an inhibitory role of MEG3 in cell proliferation (Fig. 2d). As indicated by the results of qRT-PCR and immunoblot, MEG3 overexpression led to the suppression of the mRNA and protein expressions of α-SMA and Col1A1 (Fig. 2e, f). These data suggest an inhibitory role of MEG3 in HSC activation.

### Upregulation of MEG3 suppressed liver fibrosis via EMT

**Fig. 3:**
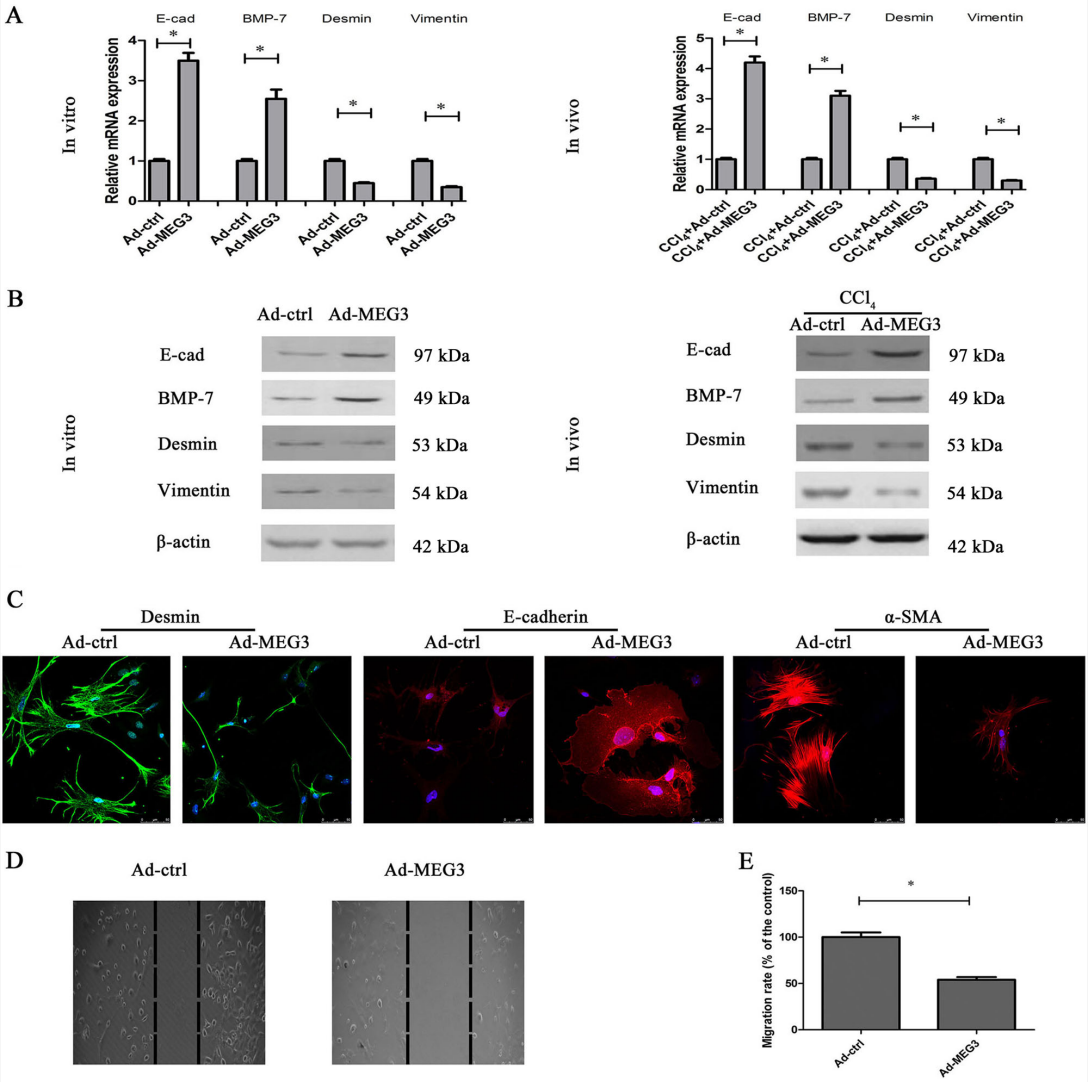
Effects of MEG3 overexpression on EMT process. Primary HSCs at Day 0 were transduced with Ad-MEG3 for 48 h. The mRNA (**a**) and protein (**b**) expressions of E-cadherin, BMP-7, Desmin and Vimenin were detected in MEG3 over-expressing HSCs (in vitro) and in the livers of CCl_4_ mice after Ad-MEG3 treatment (in vivo). **c** Immunofluorescence staining for Desmin (green), E-cadherin (red), and α-SMA (red) were evaluated by confocal laser microscopy. DAPI stained nuclei blue. Scale bar, 50 μm. **d** Cell migration was examined by wound healing. Dashed line indicates edge of cell migration. **e** Cell migration was evaluated by transwell migration assay. Five fields of migrated cells in the lower side of transwell were counted with a microscope at ×100. ^*^P < 0.05

To explore whether EMT process takes a part in the roles of MEG3 in HSC activation, we examined EMT markers including E-cadherin and BMP-7 (epithelial marker), and Desmin and Vimenin (mesenchymal marker). qRT-PCR analysis showed that Ad-MEG3 induced an increase in the mRNA expressions of E-cadherin and BMP-7 and caused a reduction in the mRNA expressions of Desmin and Vimentin in vitro as well as in vivo (Fig. 3a). In line with these, the results of immunoblot in vitro as well as in vivo confirmed an inhibitory role of MEG3 in EMT process, with an increase in the protein expressions of E-cadherin and BMP-7 and a reduction in the protein expressions of Desmin and Vimentin (Fig. 3b). Accordingly, EMT process was inhibited by MEG3 overexpression in isolated primary HSCs from CCl_4_ mice (Fig. S3B). To further confirm it, immunofluorescence analysis was performed in MEG3-overexpressing HSCs. Overexpression of MEG3 resulted in an increase in E-cadherin as well as a reduction in Desmin and α-SMA (Fig. 3c). Using wound healing and transwell migration asssays, the effects of MEG3 on cell migration were explored. Upregulation of MEG3 resulted in the suppression of HSC migration (Fig. 3d, e). Next, effects of downregulation of MEG3 on EMT process were explored. Adenoviral vectors expressing shRNA against MEG3 (Ad-shMEG3) treatment induced a significant reduction in MEG3 expression (Fig. S4A). Ad-shMEG3 treatment caused an increase in Desmin and Vimentin as well as a reduction in E-cadherin and BMP-7 (Fig. S4B). These results suggest that MEG3 contributes to the suppression of liver fibrosis, at least in part, via suppressing EMT. Whether MEG3 is involved in EMT process in hepatocytes was next examined. We demonstrated that EMT process was inhibited in primary hepatocytes isolated from CCl_4_ mice after Ad-MEG3 treatments, with an increase in E-cadherin and a reduction in Vimentin (Fig. S3C and Fig. S3D). Our data suggest that MEG3 is involved in hepatocyte EMT process.

### Hh pathway is suppressed by MEG3 overexpression

**Fig. 4:**
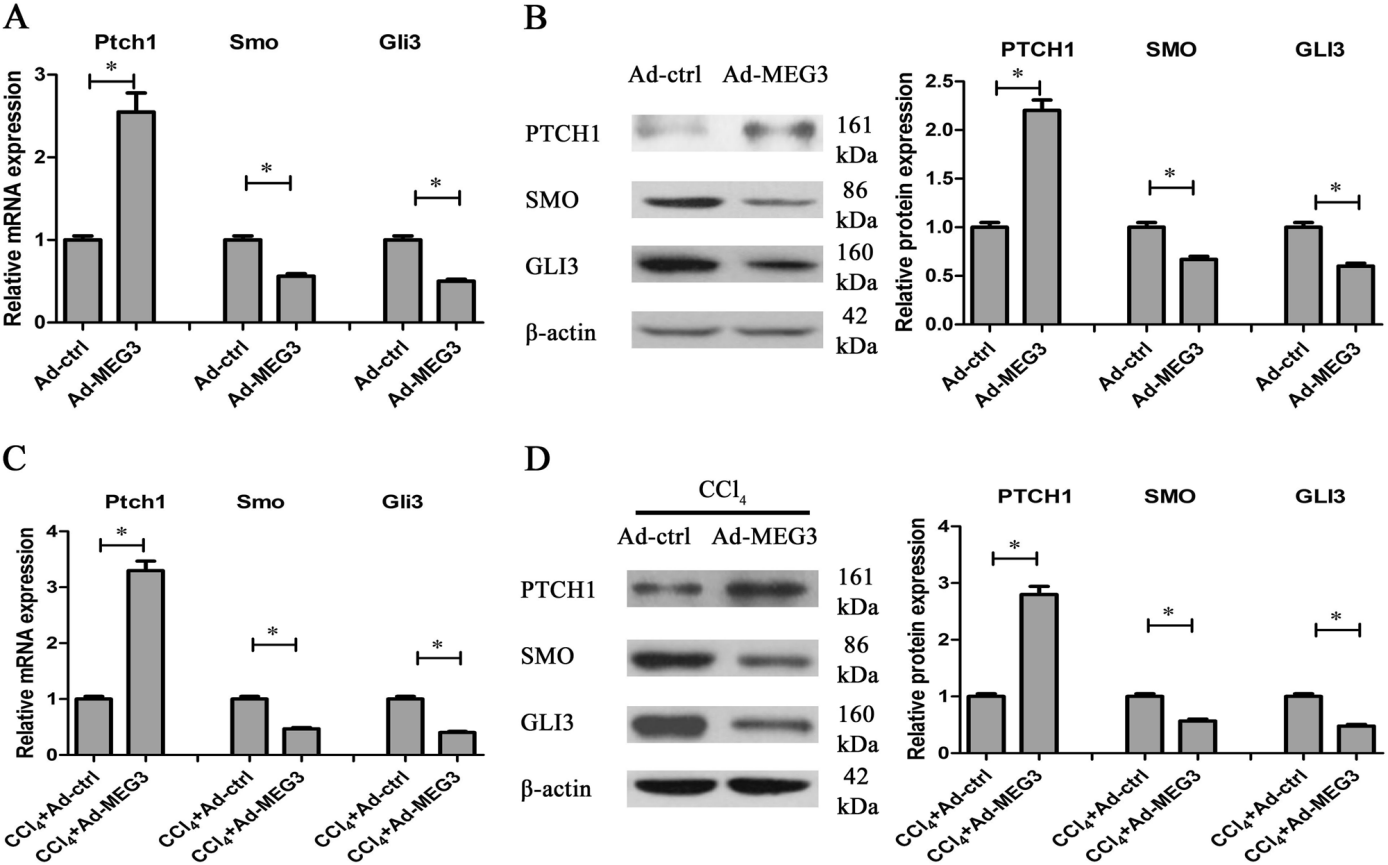
Effects of MEG3 on Hh pathway. Primary 4-day-old HSCs were transduced with Ad-MEG3 for 48 h. **a** In HSCs, Ptch1 mRNA was enhanced by MEG3, whereas the mRNA expressions of Smo and Gli3 were reduced by MEG3. **b** In HSCs, MEG3 induced an increase in Ptch1 protein and a reduction in the protein expressions of Smo and Gli3. **c** In CCl_4_ mice, Ad-MEG3 treatment enhanced Ptch1 mRNA and reduced the mRNA expressions of Smo and Gli3. **d** In CCl_4_ mice, Ad-MEG3 treatment resulted in an increase in Ptch1 protein and a decrease in the protein expressions of Smo and Gli3. ^*^P < 0.05

It has been reported that activated EMT process promotes HSC activation and liver fibrosis through activating Hh pathway^5,6^. Next, we explored whether Hh pathway plays a vital role in MEG3 over-expressing HSCs. qRT-PCR and immunoblot analysis were used to detect Hh-related genes, including Ptch1, Smo, and Gli3. In primary HSCs, MEG3 overexpression resulted in an increase in Ptch1 as well as a reduction in Smo and Gli3 (Fig. 4a, b). Similarly, MEG3 suppressed the activation of Hh pathway in vivo, including increased Ptch1 as well as reduced Smo and Gli3 (Fig. 4c, d). By contrast, Ad-shMEG3 treatment led to a reduction in expression of Ptch1 and an increase in expressions of Smo and Gli3 (Fig. S4C). Taken together, we demonstrate that MEG3 inhibits EMT process, at least in part, via suppressing Hh pathway.

### MEG3 interacts with SMO

**Fig. 5:**
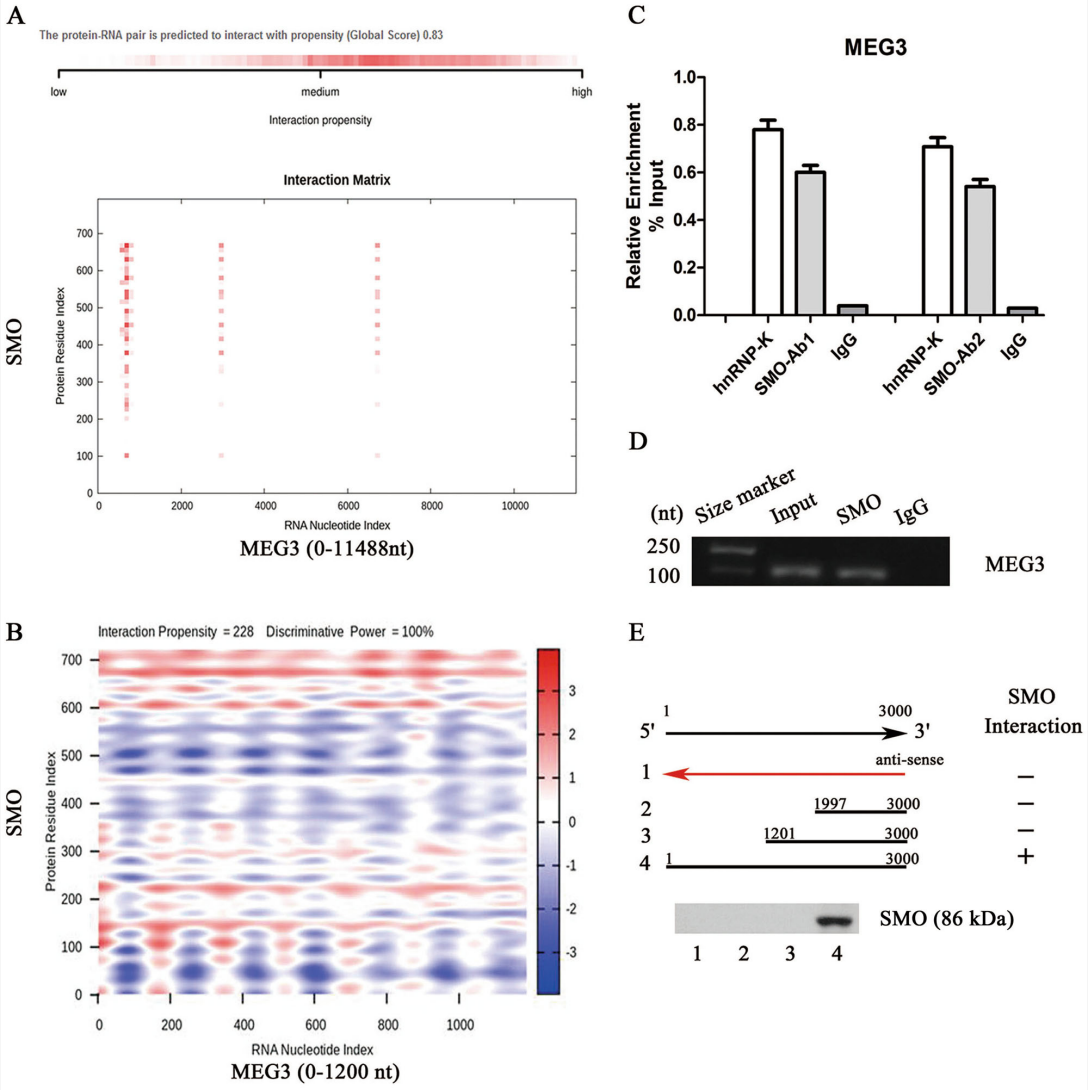
MEG3 physically interacts with SMO protein. **a** The overall interaction propensity of MEG3 and SMO protein was predicted by catRAPID. **b** Predicted interaction between MEG3 (nucleotide positions 0–1200 nt) and SMO protein (amino acid residues 0–720). **c** RIP experiments were performed using SMO antibody in primary HSCs at Day 0. qRT-PCR was performed to detect pulled-down MEG3. hnRNP-K antibody and IgG were used as positive and negative controls, respectively. **d** SMO asscociated MEG3 was detected by regular RT-PCR. **e** Mapping the SMO interaction region of MEG3. Biotinylated RNAs corresponding to different fragments of MEG3 or its antisense sequences (red line) were co-incubated with cell lysates and associated SMO proteins were detected by immunoblotting

Recently, a new regulatory mechanism of lncRNAs in modulating diverse biological processes is through interacting with RNA binding proteins. For example, lincRNA-p21 enhances p53 transcriptional activity in atherosclerosis via binding MDM2, an E3 ubiquitin–protein ligase^21^. Due to Hh pathway was inactivated by MEG3, we explored the possible binding between Hh-related proteins and MEG3. The binding of protein–RNA was predicted by bioinformatic analysis (catRAPID)^22^. Our results showed that MEG3 may bind with SMO (Fig. 5a). However, other Hh-related proteins such as PTCH1, GLI1, GLI2, and GLI3 were predicted no interaction with MEG3 (data not shown). As shown by Fig. 5a, the propensity of the overall interaction between SMO and MEG3 was very high. It was found that nt 0–1200, 2500–3300, or 6600–7100 of MEG3 was predicted to interact with SMO. Notably, the binding propensity of nt 0–1200 position of MEG3 was higher than other positions of MEG3 (Fig. 5a). Analysis of catRAPID further confirmed the interaction between 0 and 1200 position of MEG3 and SMO, with discriminative power of 100% (Fig. 5b). Therefore, nt 0–1200 of MEG3 was selected to the next experiment. Then, RNA immunoprecipitation (RIP) was performed to detect the direct binding of MEG3 and SMO in primary HSCs at Day 0 isolated from healthy mice. Two independent anti-SMO antibodies were applied to suggest the specificity of the interaction. Analysis of RIP experiments suggested the interaction between MEG3 and SMO (Fig. 5c, d). Deletion-mapping results demonstrated that nt 0–1200 of MEG3 interacted with SMO protein (Fig. 5e). In contrast, there was no interaction between the nt 1201–11488 region of MEG3 and SMO, as confirmed by deletion-mapping analysis (data not shown). RIP experiments were additionally performed in primary HSCs isolated from CCl_4_ mice with Ad-MEG3 treatment. As shown in Fig. S5A, there was a significant increase in MEG3 enrichment in CCl_4_ mice with Ad-MEG3 treatment compared with CCl_4_ mice with adenoviral vectors expressing a control scrambled sequence (Ad-Ctrl) treatment. These data suggest that the binding of MEG3 with SMO protein was enhanced by MEG3 overexpression in vivo. To further determine the importance of the binding between MEG3 and SMO, these binding sites were mutated (Ad-MEG3-Mut). Interestingly, Ad-MEG3-Mut almost blocked down MEG3 overexpression-suppressed Gli3 expression and EMT process in HSC activation (Fig. S5B and Fig. S5C). The results suggest that the interaction between MEG3 and SMO is involved in the suppression of EMT caused by MEG3 overexpression.

### MEG3 is a target of microRNA-212 (miR-212)

Interestingly, lack of the SMO binding site in MEG3 could not completely inhibit the effects of MEG3 on Gli3 and EMT process (Fig. S5B and Fig. S5C). It is possible that MEG3 inhibits EMT process in HSC activation via multiple mechanisms. Recent studies showed that lncRNAs serve as competing endogenous RNAs (ceRNAs) to sponge miRNAs and then contribute to the derepression of miRNA targets^12^. Previously, miRNAs such as miR-212 and miR-17-5p have been reported to be involved in liver fibrosis progression^10,23,24^. We next analyzed whether they are involved in the effects of MEG3 on liver fibrosis. As shown in Fig. S6A, there was a significant reduction in miR-212 in cells with MEG3 overexpression and miR-212 was selected for the next experiment. It was found that miR-212 was increased during liver fibrosis in vivo and in vitro (Fig. S6B). Using bioinformatic analysis (Targetscan), it was found that Ptch1 was a potential target of miR-212 (Fig. S6C). Luciferase acitivity assays showed that miR-212 inhibited luciferase acitivity of pmirGLO-Ptch1-Wt without affecting that of pmirGLO-Ptch1-Mut (Fig. S6C). In addition, miR-212 mimics induced a reduction in Ptch1 mRNA level (Fig. S6D). These data suggest that miR-212 targets Ptch1. Moreover, an interaction between MEG3 and miR-212 was predicted by RNA22 software. We generated a MEG3 luciferase reporter containing miR-212 sites (MEG3-Wt) or mutated sites (MEG3-Mut). We found that miR-212 induced a reduction in luciferase acitivity of MEG3-Wt and had no effect on luciferase acitivity of MEG3-Wt (Fig. S6E). The interaction between MEG3 and miR-212 was further confirmed by RNA pull down (Fig. S6F). Notably, MEG3-increased Ptch1 expression was inhibited by miR-212 (Fig. S6G). The similar results were seen in Smo expression, suggesting an inhibitory role of miR-212 in Hh pathway. Accordingly, MEG3-inhibited EMT process could be partly suppressed by miR-212 (Fig. S6H). Our data suggest that miR-212-mediated Hh pathway is involved in the effects of MEG3 on EMT process.

### Downregulation of MEG3 expression in CHB patients is associated with fibrosis stages

**Fig. 6:**
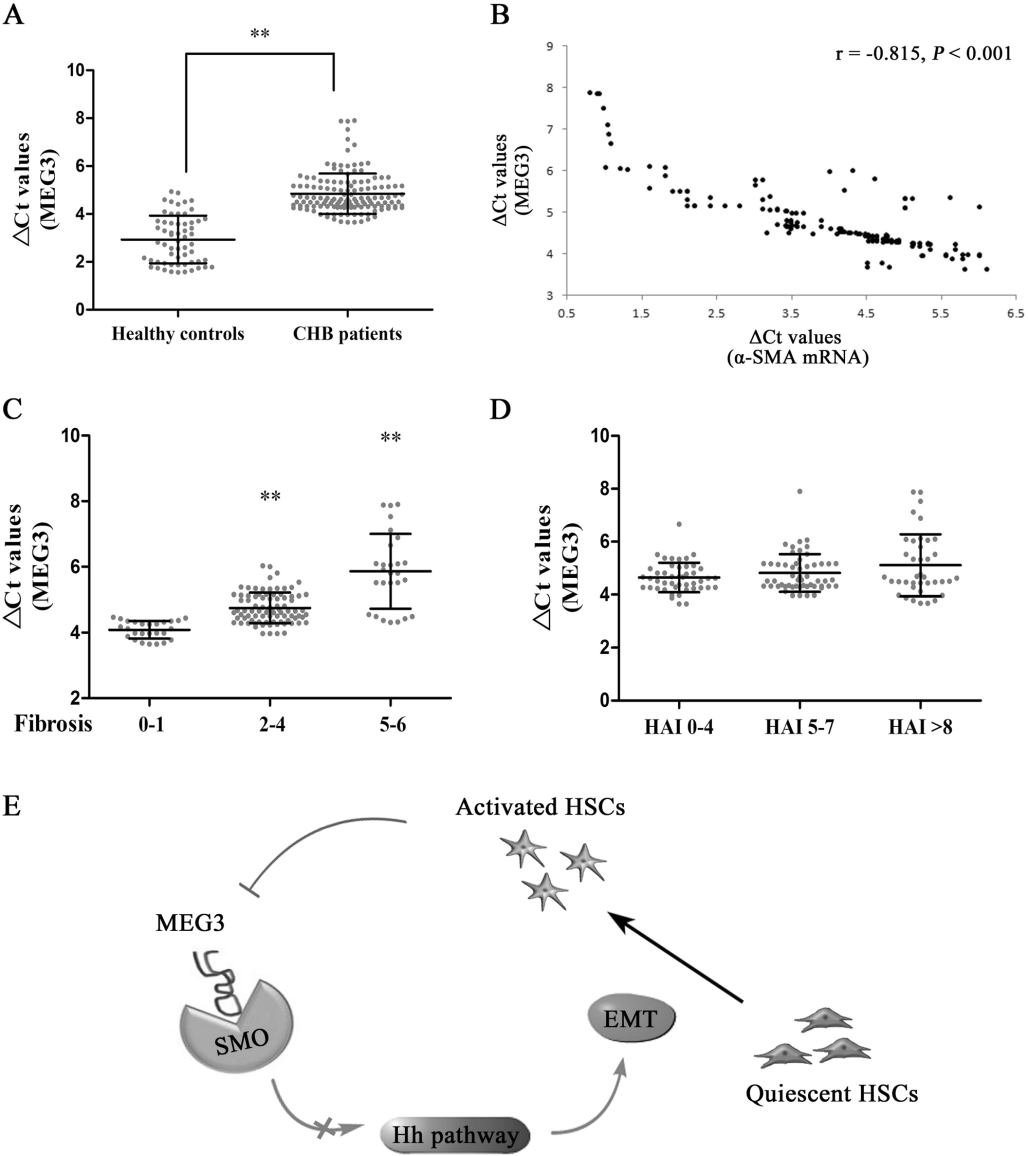
Downregulation of MEG3 expression in liver correlates with fibrosis stage in CHB patients. **a** Liver MEG3 was downregulated in CHB patients. **b** Negative correlation between transcriptional level of α-SMA and MEG3 in fibrotic human liver tissues. Pearson’s correlation analysis was used for statistical analysis. **c** △Ct values of MEG3 levels in CHB patients with fibrosis score 0–1, fibrosis score 2–4, and fibrosis score 5–6. **d** △Ct values of MEG3 levels in CHB patients with HAI score 0–4, HAI score 5–7, and HAI score >8. **e** Schematic representation of a working model by which MEG3 suppresses the activation of Hh pathway via binding to Smo. The △Ct method was used to calculate MEG3 expression, which was normalized to GAPDH, and smaller ΔCt value indicated higher expression

Finally, we sought to determine whether aberrantly expressed MEG3 is present in chronic hepatitis B (CHB) patients with liver fibrosis. Totally, 139 CHB patients and 60 healthy controls were recruited in this study (Table S1). Between CHB group and control group, no significant differences were found in age (*P* = 0.578) as well as sex distribution (*P* = 0.412, *χ*^2^ test). qRT-PCR analysis showed that liver MEG3 was lower in CHB patients than that in healthy controls (Fig. 6a). As shown by Fig. 6b, there was a negative correlation between MEG3 and the transcriptional level of α-SMA (*r* = −0.815, *P* < 0.001). Interestingly, MEG3 positively correlated with E-cadherin mRNA expression (*r* = 0.799, *P* < 0.001, Fig. S7A). Next, the association between liver MEG3 and fibrosis stages was further explored. According to their fibrosis scores, all CHB patients were divided into groups as following: low-score group (0–1), medium-score group (2–4), and high group (5–6). Increasing fibrosis scores were accompanied by a progressive increase in ΔCt values of liver MEG3, indicating that a negative correlation MEG3 expression and fibrosis scores (Fig. 6c). However, there was no correlation between MEG3 expression and histological activity index (HAI) scores (Fig. 6d). To further confirm whether MEG3 is a useful biomarker in liver fibrosis, MEG3 expression was examined in patients with alcoholic cirrhosis. Our data also indicated a reduction in MEG3 expression in patients with alcoholic cirrhosis compared with healthy controls (Fig. S7B). Our results indicate that MEG3 may be a potential biomarker in liver fibrosis.

## Discussion

MEG3, expressed in many human normal tissues, is often downregulated in various human cancers^14,25,26^. It has been demonstrated that restoring of MEG3 expression may contribute to the suppression of tumor cell proliferation and the induction of cell apoptosis^15^. Therefore, MEG3 functions as a tumor suppressor. The inhibitory role of MEG3 was also found in liver fibrosis. He et al.^18^ previously demonstrated that MEG3 suppresses liver fibrosis through p53. In addition, their double immunofluorescence stainings demonstrated that MEG3 was primarily co-localized with α-SMA expression, suggesting that HSCs may be one of the main sources of the MEG3 levels present in CCl_4_-treated livers. In lined with their results, we found that higher MEG3 expression was found in qHSCs compared with hepatocytes. By contrast, MEG3 was reduced in aHSCs in comparison with hepatocytes. In this study, MEG3 was shown to be reduced during HSC activation. Previously, the inhibitory role of MEG3 in liver fibrosis was mainly confirmed in vitro^18^. Notably, our results showed that restoring of MEG3 contributed to the suppression of liver fibrosis both in vitro and in vivo. We demonstrate that MEG3 inhibits liver fibrosis, at least in part, via suppressing Hh-mediated EMT process. In addition, we reveal that MEG3-mediated EMT process is through SMO protein and miR-212.

The crucial roles of MEG3 in regulation of EMT process have been reported in cancers^17,27^. In this study, the roles of MEG3 in regulation of EMT process in activation of HSCs were explored. Obviously, EMT process during HSC activation was suppressed by MEG3. Recently, Hh pathway-mediated EMT process in HSC activation has been shown to be involved in liver fibrosis^5,6^. Our data suggest that activation of Hh pathway was inhibited by MEG3 in liver fibrosis. Notably, using bioinformatic analysis, there may be an interaction between MEG3 and SMO. It was next confirmed by RIP assays. As shown by deletion-mapping analysis, nt 0–1200 of MEG3 was demonstrated to interact with SMO protein. Notably, lack of the SMO binding site in MEG3 almost blocked down MEG3 overexpression-suppressed Gli3 expression and EMT process in HSC activation. We demonstrate that MEG3 inhibits Hh-mediated EMT, at least in part, through interacting with SMO, which is a novel mechanism in regulation of liver fibrosis (Fig. 6e). Taken together, our results suggest an antifibrotic role of MEG3 in liver fibrosis and this is a first report to show MEG3-mediated EMT process in liver fibrosis via SMO protein. Moreover, we explored the roles of MEG3 in EMT in hepatocytes. Previously, Zeisberg et al.^28^ suggested hepatocytes-undergoing EMT as a source of myofibroblasts, contributing to liver fibrosis in vivo. In this study, we found that EMT process was inhibited in primary hepatocytes isolated from CCl_4_ mice after Ad-MEG3 treatment, with an increase in E-cadherin and a reduction in Vimentin. Our data suggest that MEG3 could suppress hepatocyte EMT process. Our results further confirm an inhibitory role of MEG3 in liver fibrosis.

Recent studies have shown that MEG3 could act as a ceRNA to regulate disease progression. Yan et al. found that MEG3 regulates ischemic neuronal death by targeting miR-21/PDCD4 signaling pathway^29^. Due to the reason that lack of the SMO binding site in MEG3 could not completely inhibit the effects of MEG3 on Gli3 and EMT process, it is possible that MEG3 inhibits EMT process in HSC activation via multiple mechanisms. In this study, miR-212 was obviously reduced in MEG3 over-expressing cells and Ptch1, a negative regulator factor of Hh pathway, was a target of miR-212. Finally, we revealed that MEG3 could regulate Hh pathway activation via miR-212, and this is why MEG3 could induce an increase in Ptch1 and a reduction in Smo. We also demonstrated that MEG3 could sponge miR-212, which may be partly responsible for the effects of MEG3 on EMT process.

Importantly, the feasibility of using liver MEG3 as a biomarker for patients with liver fibrosis was also investigated. Compared with healthy controls, liver MEG3 was obviously downregulated in CHB patients as well as patients with alcoholic cirrhosis. Increasing evidence shows that downregulation of MEG3 may be caused by its promoter methylation^14,18,30^. Next, we found that MEG3 level did not correlate with HAI scores, indicating that MEG3 may not represent a marker of necroinflammation in CHB patients. Further studies showed that MEG3 level negatively correlates with fibrosis stage in CHB patients. There was additionally a negative correlation between MEG3 level and α-SMA expression, suggesting liver MEG3 may be a useful biomarker for monitoring liver fibrosis progression in CHB patients. Moreover, MEG3 positively correlates with E-cadherin expression in CHB patients, which is consistent with the results in mice. However, the clinical significance of liver MEG3 in CLDs patients including chronic hepatitis C patients needs further verification and analysis in large samples.

In conclusion, we demonstrate that MEG3 inhibits Hh-mediated EMT process in HSC activation via interacting with SMO protein and sponging miR-212. Our results also suggest the feasibility of MEG3 as a potential biomarker in CHB patients.

## Materials and methods

### Human specimens

Participants including 60 healthy controls and 139 CHB patients undergoing liver biopsy were recruited in the First Affiliated Hospital of Wenzhou Medical University. For CHB patients, liver fibrosis was diagnosed by liver biopsy. Written informed consent was obtained from participants before liver biopsy. This project, which was approved by the Ethics Committee of the First Affiliated Hospital of Wenzhou Medical University, was in accordance with the Declaration of Helsinki.

### Liver histology

Each liver specimen was obtained at least in 2.0 cm in length after liver biopsy performance by the use of a 16-gauge Menghini needle. Then, samples were performed with the staining of haematoxylin–eosin and at least 8–10 portal tracts should be found in these specimens. Using the Ishak scoring system, the levels of HAI and fibrosis stages (F0 = no fibrosis and F6 = cirrhosis) were evaluated by the experienced hepatopathologists^31^.

### Isolation and culture of primary HSCs and hepatocytes

Primary HSCs were isolated as described previously and cultured with DMEM^32^. α-SMA immunocytochemical staining was performed to assess the purity of culture and the purity reached >98%. Hepatocytes were isolated using a two-step collagenase perfusion technique^33^. qRT-PCR was performed to examine the levels of F4/80, CD32b and CYP3A11 to assess the hepatocyte purity and the purity reached >95%.

### CCl_4_ liver injury model

Eight week-old male C57BL/6 J mice were given a biweekly intraperitoneal dose of a 10% solution of CCl_4_ (Sigma-Aldrich) in olive oil (7 μl/g/mouse) for 8 weeks. Control mice group was made by olive oil using the same method. Mice were sacrificed under anesthesia and the obtained livers were used to Masson staining analysis.

### Ad-MEG3 treatment in vivo

CCl_4_ (diluted 1:9 in olive oil) or vehicle (olive oil) was administered by intraperitoneal injection at a dose of 7 mL/kg of body weight two times weekly for six weeks to induce liver fibrosis. During CCl_4_ treatment, Ad-MEG3 (1 × 10^9^ pfu/100 μL) was injected into mice every two weeks by way of the tail vein for six weeks. All the mice were randomly divided into groups as following: olive oil (*n* = 6), CCl_4_ (*n* = 6), CCl_4_ plus Ad-Ctrl (*n* = 6) and CCl_4_ plus Ad-MEG3 (*n* = 6). Mice were sacrificed under anesthesia and the obtained livers were used to further analysis such as Sirius Red staining and hydroxyproline.

### qRT-PCR

miRNeasy Mini Kit (Qiagen, Valencia, CA, USA) was used to extract the total RNA from primary cells as well as liver tissues. Then, ReverTra Ace qPCR RT Kit (Toyobo, Osaka, Japan) was used to reverse-transcribed total RNA to cDNA. Using SYBR Green real-time PCR Master Mix (Toyobo, Osaka, Japan), qRT-PCR was perfromed to detect gene expression. The primers of Vimentin, Desmin, E-cadherin, Ptch1, Smo, Col1A1, α-SMA, GAPDH, and human MEG3 were designed as described previously^34,35^. As shown in Supporting Table 2, the primers of other genes were designed. Expressions of miRNAs were detected using the TaqMan MicroRNA Assay (Applied Biosystems, Foster City, CA). The GAPDH level was used to normalize the relative abundance of mRNAs and MEG3. U6 level was used to normalize the relative abundance of miRNAs. The expression levels (2^−⊿⊿Ct^) of MEG3, mRNAs and miRNAs were calculated as described previously^36^.

### Immunoblot analysis

Tissues and cells were lysed with ice-cold lysis buffer (50 mM Tris-HCl, pH 7.4, 100 mM 2-mercaptoethanol, 2% w/v SDS, 10% glycerol). Total proteins were quantified and separated by SDS-PAGE. Then immunoblot assay was performed as described previously^37^. β-actin was used as internal control.

### Statistical analysis

Data from at least three independent experiments were expressed as the mean ± SD. Differences between multiple groups were evaluated using one-way analysis of variance. Differences between two groups were compared using a Student’s *t* test. The Mann–Whitney test or Kruskal–Wallis test was performed to determine the significance of liver MEG3 levels in CHB patients. *P* < 0.05 was considered significant. Correlation between α-SMA expression and MEG3 level in liver tissues was examined by Pearson’s correlation coefficient. All statistical analyses were performed with SPSS software (version 13; SPSS, Chicago, IL). Other methods are described in Supplementary materials and methods.

## Electronic supplementary material

Supplementary materials and methods

Supporting Information

Table.S1

Table.S2
