# Correction: LncRNA-MEG3 inhibits activation of hepatic stellate cells through SMO protein and miR-212

**DOI:** 10.1038/s41419-022-04558-6

**Published:** 2022-02-09

**Authors:** Fujun Yu, Wujun Geng, Peihong Dong, Zhiming Huang, Jianjian Zheng

**Affiliations:** 1 Departments of Gastroenterology and Hepatology, The First Affiliated Hospital of Wenzhou Medical University, Wenzhou, 325000 China; 2 Department of Anesthesiology, The First Affiliated Hospital of Wenzhou Medical University, Wenzhou, 325000 China; 3 Department of Infectious Diseases, The First Affiliated Hospital of Wenzhou Medical University, Wenzhou, 325000 China; 4 Key Laboratory of Diagnosis and Treatment of Severe Hepato-Pancreatic Diseases of Zhejiang Province, The First Affiliated Hospital of Wenzhou Medical University, Wenzhou, 325000 China

Correction to: *Cell Death and Disease* 10.1038/s41419-018-1068-x, published online 03 October 2018

The original version of this article unfortunately contained a mistake in Fig. 1A and Fig. 3D.

In the original Fig. 1A, an incorrect image for the α-SMA immunocytochemical staining panel was inadvertently included. As shown in α-SMA staining in Fig. 1A, the image of 4-week CCl4 mice (mice were treated CCl4 for 4 weeks) was uploaded as the CCl4 mice (CCl4 mice should be treated CCl4 for 8 weeks). In the original Fig. 3D, the cell migration image of Ad-Ctrl group was wrongly uploaded as Ad-MEG3 group. The corrected images are provided below. The authors confirm that these errors do not affect the results and conclusions of the study. The authors apologize for any inconvenience caused.

**Fig. 1:**
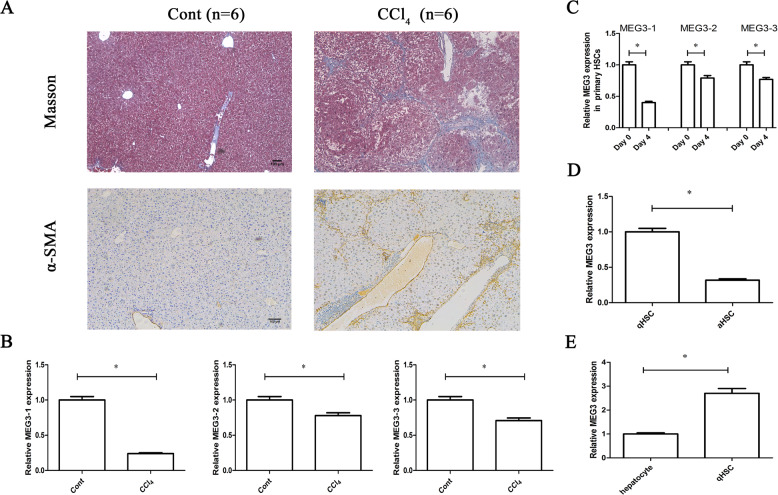
▓.

**Fig. 3:**
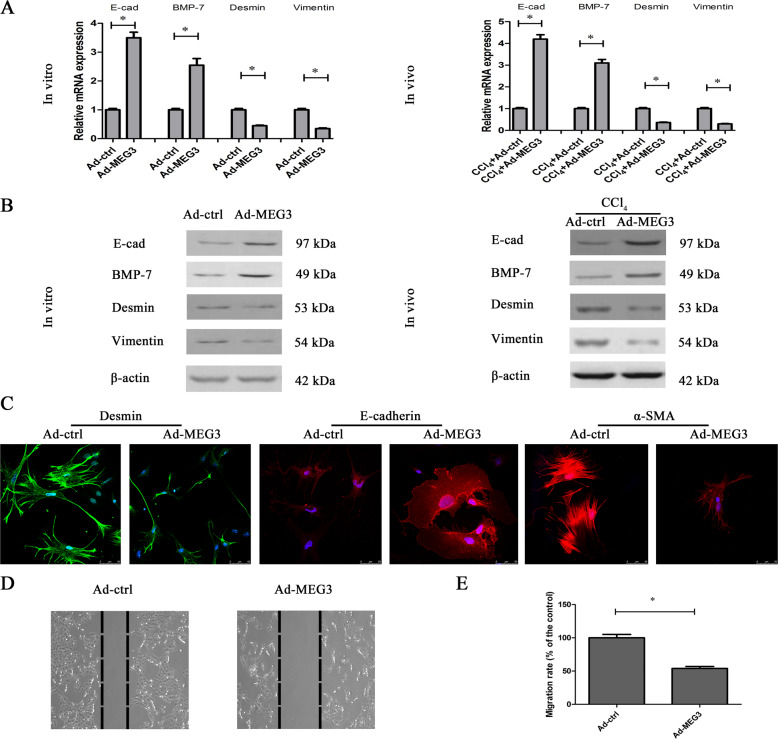
▓.

