# Supplementary material for: LncRNA-MEG3 inhibits activation of hepatic stellate cells through SMO protein and miR-212
Source: Cell Death Dis. 2018 Oct 3;9(10):1014. doi: 10.1038/s41419-018-1068-x (PMC6170498; doi:10.1038/s41419-018-1068-x)
Supplement: Supplementary file 1 — Supplementary materials and methods [file 41419_2018_1068_MOESM1_ESM.docx]

**Materials**

CCl_4_ and TGF-β1 were obtained from Sigma (St Louis, MO, USA). Antibodies against type I collagen, E-cadherin, BMP-7, Desmin, Vimentin, Ptch1, Smo, Gli3, α-SMA and β-actin were obtained from Abcam (Cam-bridge, MA, USA). Ad-MEG3, Ad-Ctrl, Ad-shMEG3 and adenoviral vectors expressing the scrambled shRNA (Ad-shCtrl) were synthesized by GenePharma biotechnology (Shanghai, China). All mice were provided by the Experimental Animal Center of Wenzhou Medical University. The animal experimental protocol was approved by the University Animal Care and Use Committee.

**Hepatic hydroxyproline content**

Liver tissues (50 mg) were homogenized in HCl and hydrolyzed at 120°C overnight. After lysate centrifugation at 12,000 g for 10 min at 4°C, the supernatant was evaporated to dryness under vacuum. The hepatic hydroxyproline content was assessed using the Hydroxyproline Colorimetric Assay kit (BioVision, San Francisco, CA). Data were normalized to liver weight.

**Immunohistochemistry**

Immunohistochemical staining was performed on the sections (3 μm thick) from the liver tissues. Briefly, after deparaffinization, hydration, and antigen retrieval, samples were incubated overnight at 4°C with a primary antibody against α-SMA (1:100) and then with a biotinylated secondary antibody. α-SMA expression was visualized by 3,3'-diaminobenzidine tetrahydrochloride (DAB) staining. Slides were counterstained with hematoxylin before dehydration and mounting α-SMA-positive areas within the fibrotic region were then observed. Quantitative analysis was calculated from five fields for each liver slice.

**Immunofluorescence microscopy**

Cells were transduced with Ad-MEG3 for 48 h, washed with PBS and fixed in an acetic acid: ethanol (1:3) solution for 5 min at -20°C. Nonspecific binding was blocked with 5% goat serum in PBS for 1 h at room temperature, and the cells were then incubated with primary antibodies against α-SMA, E-cadherin or Desmin (Abcam) in a humidified chamber. After washing twice in PBS, the cells were incubated with fluorescein-labelled secondary antibody (1:50 dilution; Dianova Hamburg, Germany) in antibody dilution solution for 1 h at room temperature in the dark. The nuclei were stained with 4,6-diamidino-2-phenylindole (DAPI) in the dark for 30 min at room temperature. The slides were washed twice with PBS, covered with DABCO (Sigma-Aldrich), and examined by confocal laser scanning microscopy (Olympus, Tokyo, Japan) at 488 or 568 nm.

**Proliferation assay**

Cell proliferation was detected by EdU asssys. Cells were transduced with Ad-MEG3 or Ad-Ctrl, and then labelled with EdU for 12 h. The proliferative rate was detected using a Cell-Light™ EdU In Vitro Imaging Detection Kit (Guangzhou RiboBio Co., Ltd., cat# C10310-1) according to the manufacturer’s instructions.

**Transwell migration assays**

Primary HSCs at Day 0 were placed in the top chamber of transwell migration chambers (8 μm; Millipore, Billerica, MA, USA). After 48 hrs, cells which had not migrated to the lower chamber were removed from the upper surface of the transwell membrane with a cotton swab. Migrating cells on the lower membrane surface were fixed, stained, photographed and counted using a microscope at ×100. Experiments were assayed in triplicate, and ≥5 fields were counted in each experiment.

**Wound healing assay**

Primary HSCs at Day 0 were cultured in a monolayer in the absence (control group) or presence (MEG3 group) of Ad-MEG3 for 48 hrs. After 48 hrs of incubation, wounds were made by scraping through the cell monolayer with a sterile 200-μl pipette tip, followed by washing with medium to remove cellular debris. Three wounds were made in the dish. Twenty-four hours after wounding, phase-contrast images at three wound sites along the scratch were examined and photographed by phase-contrast microscopy at ×100 (Nikon, Tokyo, Japan). Triplicate results were obtained in three separate experiments.

**RIP assays**

RIP experiments were performed using the Magna RIP RNA-Binding Protein Immunoprecipitation Kit (Millipore,MA, USA) following the manufacturer's instructions. Two independent Smo antibodies were used.

**Biotin RNA pull-down assay and deletion mapping and bioinformatics**

Briefly, the pcDNA3.1-MEG3 plasmid was used as template to synthesize biotinylated MEG3 transcripts. For biotinylated RNA generation, PCR products were used for in vitro transcription with the Biotin RNA Labeling Mix and T7 RNA polymerase. Nuclear proteins were extracted using Nuclear and Cytoplasmic Protein Extraction Kit (Beyotime Biotechnology, Jiangsu, China). After incubation, binding and washing, beads were boiled in SDS buffer, and retrieved protein was detected by standard western blotting.

**Pull-down assay with Bio-miR-212**
Briefly, after 48 h of HSCs transfected with Bio-miR-212-Wt, Bio-miR-212-Mut, or Bio-miR-NC, the cells were washed with PBS followed by incubation in a lysis buffer for 10 min. To exclude RNA and protein complexes, the beads were blocked in lysis buffer including RNase-free bovine serum albumin (BSA) and yeast tRNA (Sigma). After the lysates were incubated with streptavidin-coated magnetic beads (Life Technologies) at 4°C for 4 h, they were washed twice with lysis buffer, three times with the low salt buffer, and once with the high salt buffer. The bound RNAs were isolated using TRIzol reagent (Life Technologies). MEG3 expression was determined by qRT-PCR.

**Luciferase reporter assay**pmirGLO-Ptch1 or pmirGLO-MEG3 was cotransfected with miR-212 or miR-NC into HEK293T cells by lipofectamine-mediated gene transfer as described previously [12]. The relative luciferase activity was normalized to Renilla luciferase activity 48 hours after transfection.

**Liver specimens of patients with alcoholic cirrhosis**

Participants including 15 healthy controls and 15 alcoholic cirrhosis patients undergoing liver biopsy were recruited in the First Affiliated Hospital of Wenzhou Medical University. These patients had history of alcohol consumption averaging at least 80 g per day (for men) or 50 g per day (for women) for at least 10 years. The diagnosis of cirrhosis was made using radiographic imaging compatible with cirrhosis and/or history of ascites, grade 2 or higher hepatic encephalopathy and/or the presence of esophageal varices on upper gastrointestinal endoscopy, or biopsy-proven cirrhosis, with exclusion of hepatitis B or C, autoimmune liver disease, hemochromatosis, and Wilson disease. Written informed consent was obtained from participants before liver biopsy. This project, which was approved by the Ethics Committee of the First Affiliated Hospital of Wenzhou Medical University, was in accordance with the Declaration of Helsinki.
