# Supplementary material for: LncRNA-MEG3 inhibits activation of hepatic stellate cells through SMO protein and miR-212
Source: Cell Death Dis. 2018 Oct 3;9(10):1014. doi: 10.1038/s41419-018-1068-x (PMC6170498; doi:10.1038/s41419-018-1068-x)
Supplement: Supplementary file 2 — Supporting Information [file 41419_2018_1068_MOESM2_ESM.docx]

**Supporting Information**

**Fig.S1** (A) Schematic diagram of variants of mouse MEG3 transcript. Red box represents the sequence of MEG3-1. In MEG3-2, blue box represents its distinctive sequence and red box means the sequence could be found in MEG3-1. In MEG3-3, black box represents its distinctive sequence. In addition, red box means the sequence of MEG3-3 could be found in MEG3-1 and MEG3-2, and blue box means the sequence of MEG3-3 could be found in MEG3-2. (B) The expressions of MEG3-1, MEG3-2 and MEG3-3 in isolated primary HSCs from CCl_4_ mice. (C) Expressions of 15 human MEG3 transcript variants in TGF-β1-treated-LX-2 cells. LX-2 cells were treated with TGF-β1 (2 ng/ml) for 48 h. (D) MEG3 expression in primary hepatocytes and LX-2 cells. (E) MEG3 expression in primary hepatocytes and aHSCs (primary 8-day-old HSCs). **P*<0.05 and ***P*<0.01. Each value is the mean ± SD of three experiments.


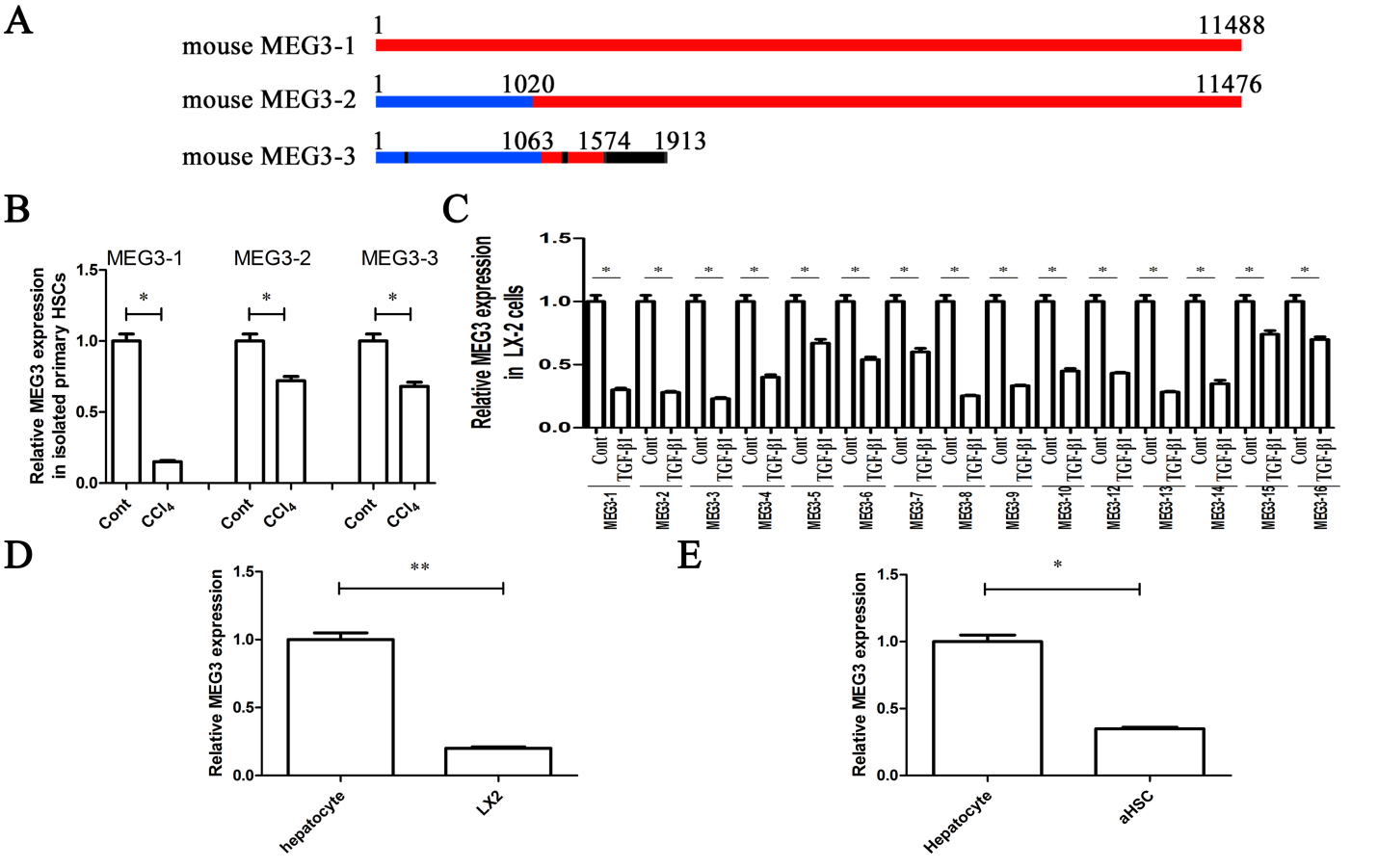


**Fig.S2** Effects of MEG3 over-expression on the expressions of MEG3, hydroxyproline, Col1A1 mRNA, ALT and AST in CCl_4_ mice. (A) MEG3 expression in the liver. (B) MEG3 expression in isolated primary HSCs. (C) hydroxyproline level. (D) Col1A1 mRNA in isolated primary HSCs. (E) ALT value. (F) AST value. **P*<0.05 compared to the control. Each value is the mean ± SD of three experiments.

**
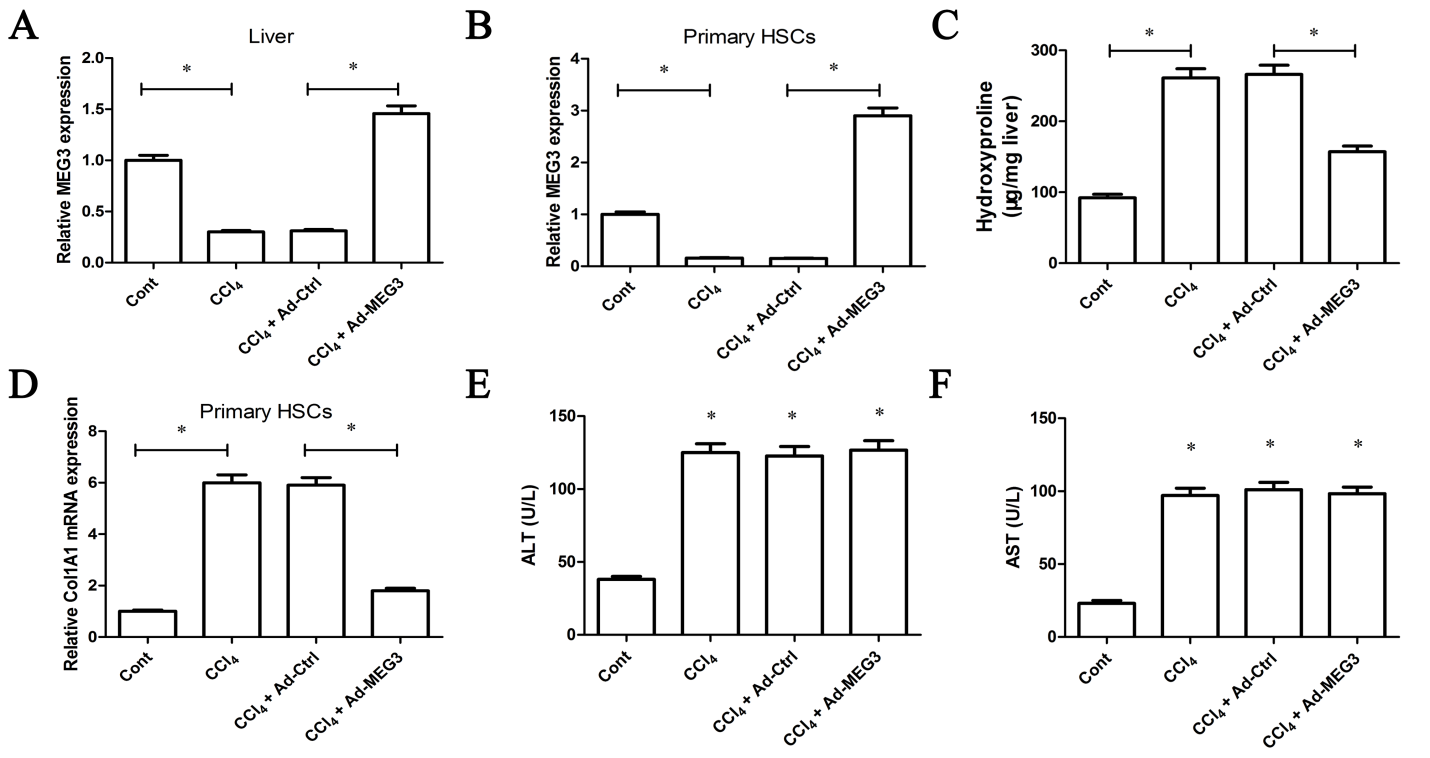
**

**Fig.S3** MEG3 over-expression inhibits EMT process. Primary HSCs and hepatocytes were isolated from CCl_4_ mice after Ad-MEG3 treatment. (A) MEG3 expression. Primary HSCs at Day 0 were transduced with Ad-MEG3 for 48 h. (B) EMT markers in isolated primary HSCs from CCl_4_ mice after Ad-MEG3 treatment. (C and D) EMT markers in isolated primary hepatocytes from CCl_4_ mice after Ad-MEG3 treatment. **P*<0.05. Each value is the mean ± SD of three experiments.


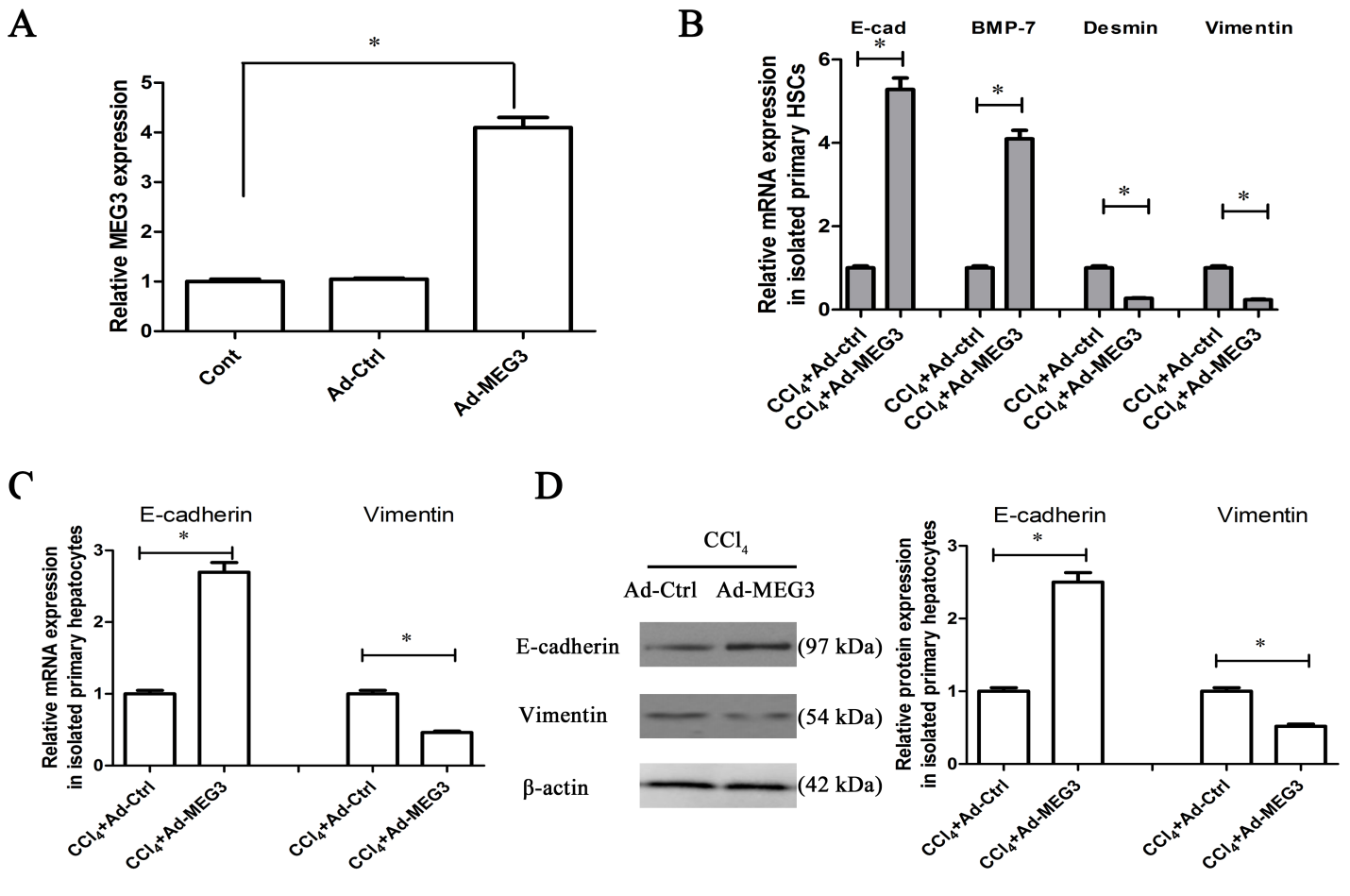


**Fig.S4** Effects of down-regulation of MEG3 on EMT process and Hh pathway *in vitro*. (A) MEG3 expression. Primary HSCs at Day 0 were transduced with Ad-shMEG3 for 48 h. (B) EMT markers. Primary HSCs at Day 0 were transduced with Ad-shMEG3 for 48 h. (C) Hh pathway markers. Primary 4-day-old HSCs were transduced with Ad-shMEG3 for 48 h. **P*<0.05. Each value is the mean ± SD of three experiments.





**Fig.S5** MEG3 interacts with SMO protein. (A) RIP experiments were performed in primary HSCs using SMO antibody. Primary HSCs were isolated from CCl_4_ mice after Ad-Ctrl or Ad-MEG3 treatment. qRT-PCR was performed to detect pulled-down MEG3. hnRNP-K antibody and IgG were used as positive and negative controls, respectively. (B) Effects of Ad-MEG3-Mut on EMT process in primary HSCs at Day 0. (C) Effects of Ad-MEG3-Mut on Gli3 mRNA in primary HSCs at Day 4. **P*<0.05 compared to the Ad-Ctrl group and ^#^*P*<0.05 compared to the Ad-MEG3 group. Each value is the mean ± SD of three experiments.





**Fig.S6** MEG3 inhibits Hh pathway activation via sponging miR-212. Primary 4-day-old HSCs were transduced with Ad-MEG3 for 48 h. (A) Expressions of miRNAs involved in liver fibrosis were detected. (B) miR-212 level was detected in CCl_4_ mice or activated HSCs. (C) Schematic diagram of the miR-212 binding site in Ptch1 based on Targetscan. Relative luciferase activities of luciferase reporters harboring the wild-type or mutant Ptch1 were analyzed 48 h following transfection with miR-212 mimics or miR-NC. (D) Ptch1 mRNA in primary HSCs transfected with miR-212 mimics for 48 h. (E) Schematic diagram of the miR-212 binding site in MEG3 based on RNA22 software. Relative luciferase activities of luciferase reporters harboring the wild-type or mutant MEG3 were analyzed 48 h following transfection with miR-212 mimics or miR-NC. (F) Pull down assay to validate the direct interaction between MEG3 and miR-212. Bio-miR-NC is not complementary to MEG3. (G) The mRNA levels of Pthc1 and Smo. Primary 4-day-old HSCs were transduced with Ad-MEG3 for 48 h and tansfected with miR-212 for additional 48 h. (H) The mRNA levels of E-cad and Vimentin. Primary 0-day-old HSCs were transduced with Ad-MEG3 for 48 h and tansfected with miR-212 for additional 48 h. **P*<0.05 compared with the control and ^#^*P*<0.05 compared with the Ad-MEG3 group. Each value is the mean ± SD of three experiments.





**Fig.S7** MEG3 correlates with E-cadherin in CHB patients and is down-regulated in patients with alcoholic cirrhosis. (A) Positive correlation between transcriptional level of E-cadherin and MEG3 in CHB patients. Pearson’s correlation analysis was used for statistical analysis. (B) △Ct values of MEG3 levels in patients with alcoholic cirrhosis. △Ct method was used to calculate MEG3 expression, which was normalized to GAPDH, and smaller ΔCt value indicated higher expression. ***P*<0.01.
