# Supplementary material for: LncRNA-MEG3 inhibits activation of hepatic stellate cells through SMO protein and miR-212
Source: Cell Death Dis. 2018 Oct 3;9(10):1014. doi: 10.1038/s41419-018-1068-x (PMC6170498; doi:10.1038/s41419-018-1068-x)
Supplement: Supplementary file 3 — Table.S1 [file 41419_2018_1068_MOESM3_ESM.doc]

**Table S1.** Patient Characteristics

| Parameter | CHB patients | healthy subjects |
| --- | --- | --- |
| *Epidemiology* |  |  |
| Gender, m/f (%) | 81/58 (58.3/41.7) | 32/28 (53.3/46.7) |
| Age, years, median (range) | 45.2 (35.2-55.8) | 46.4 (36.4-57.4) |
| *Virology* |  |  |
| HBe antigen positive, n (%) | 35 (25.2%) |  |
| HBe antigen negative, n (%) | 104 (74.8%) |  |
| *ALT* |  |  |
| Elevated ALT* | 107 (76.9%) |  |
| Normal ALT | 32 (23.1%) |  |
| *Fibrosis stage (Ishak)* |  |  |
| F0, n (%) | 12 (8.6%) |  |
| F1, n (%) | 14 (10.1%) |  |
| F2, n (%) | 30 (21.6%) |  |
| F3, n (%) | 24 (17.2%) |  |
| F4, n (%) | 32 (23.0%) |  |
| F5, n (%) | 13 (9.4%) |  |
| F6, n (%) | 14 (10.1%) |  |
| *HAI* |  |  |
| 2, n (%) | 11 (7.9%) |  |
| 3, n (%) | 20 (14.4% ) |  |
| 4, n (%) | 15 (10.8%) |  |
| 5, n (%) | 15 (10.8%) |  |
| 6, n (%) | 18 (12.9% ) |  |
| 7, n (%) | 21 (15.1% ) |  |
| 8, n (%) | 11 (7.9%) |  |
| 9, n (%) | 13 (9.4% ) |  |
| ≥11, n (%) | 15 (10.8%) |  |

* >40 U/L.
