# Supplementary material for: LncRNA-MEG3 inhibits activation of hepatic stellate cells through SMO protein and miR-212
Source: Cell Death Dis. 2018 Oct 3;9(10):1014. doi: 10.1038/s41419-018-1068-x (PMC6170498; doi:10.1038/s41419-018-1068-x)
Supplement: Supplementary file 4 — Table.S2 [file 41419_2018_1068_MOESM4_ESM.docx]

**Table.S2 Primer sequences**

| Gene | Forward sequence | Reverse sequence |
| --- | --- | --- |
| mouse MEG3-1 | 5'-GTGGACAATGGTGTCCAGGC-3' | 5'-TTAACTCAGAGCGGGTCTCC-3' |
| mouse MEG3-2 | 5'- CACCTCTACCTCCTGAGCCA-3' | 5'- TAGGGCACTGGTTCAAGGTTT-3' |
| mouse MEG3-3 | 5'-GACCCAAGACTCTGGACCCT-3' | 5'-GACACAACAGCCTTTCTCCCA-3' |
| mouse GLI3 | 5'-TGAGTCCTCACAGAGCAAGC-3' | 5'- TTGTTCCTTCCGGCTGTTCC-3' |
| mouse BMP-7 | 5'-GCTGGCAGGACTGGATCATT-3' | 5'- TGGGTACTGTGTCTGGGTTG-3' |
